# Music Publications

**Published:** 1878-02

**Authors:** 


*Music Publications*:—

From time to time we have received from F. W. Helmick, of 50 W. Fourth St., Cincinnati, Ohio, beautiful pieces of music adapted for the piano or organ. Some ten pieces are sent for $1.00, which is a remarkably low price.

